# Analysis of the Application Efficiency of TensorFlow and PyTorch in Convolutional Neural Network

**DOI:** 10.3390/s22228872

**Published:** 2022-11-16

**Authors:** Ovidiu-Constantin Novac, Mihai Cristian Chirodea, Cornelia Mihaela Novac, Nicu Bizon, Mihai Oproescu, Ovidiu Petru Stan, Cornelia Emilia Gordan

**Affiliations:** 1 Department of Computers and Information Technology, Electrical Engineering and Information Technology Faculty, University of Oradea, 410087 Oradea, Romania; 2 Department of Electrical Engineering, Electrical Engineering and Information Technology Faculty, University of Oradea, 410087 Oradea, Romania; 3 Department of Electronics, Computers and Electrical Engineering, Faculty of Electronics, Telecommunication, and Computer Science, University of Pitesti, 110040 Pitesti, Romania; 4 Department of Automation, Faculty of Automation and Computer Science, Technical University of Cluj-Napoca, 400114 Cluj-Napoca, Romania; 5 Department of Electronics and Telecommunications, Electrical Engineering and Information Technology Faculty, University of Oradea, 410087 Oradea, Romania

**Keywords:** convolutional neural network, TensorFlow, PyTorch, network training, network design

## Abstract

In this paper, we present an analysis of important aspects that arise during the development of neural network applications. Our aim is to determine if the choice of library can impact the system’s overall performance, either during training or design, and to extract a set of criteria that could be used to highlight the advantages and disadvantages of each library under consideration. To do so, we first extracted the previously mentioned aspects by comparing two of the most popular neural network libraries—PyTorch and TensorFlow—and then we performed an analysis on the obtained results, with the intent of determining if our initial hypothesis was correct. In the end, the results of the analysis are gathered, and an overall picture of what tasks are better suited for what library is presented.

## 1. Introduction

In the last couple of years, the field of Artificial Intelligence has seen numerous advancements, now arriving to the point where previously impossible tasks, like fully autonomous cars or even AI modeling in medicine [1], have become tangible objectives and have even been successfully tested. As an example, vaccines usually take years to develop, but during the COVID-19 pandemic, vaccine trials were already being performed after only a few months from the initial outbreak. This speedup is attributed to the novel ways through which the scientists combined artificial intelligence with modern study methodologies, and thus analysis and prediction tasks that would otherwise take days or weeks were reduced to mere minutes. A deeper analysis regarding some of the methods was performed by Akpofure A. Enughwure et al. [2] during the early stages of the pandemic and involved a series of reviews that targeted various applications of AI in three main categories of study: diagnosis of COVID-19, predictions on how the infection will spread, and development of medication. While some methods had more success than others, the authors reached the conclusion that integrating with artificial intelligence had the potential to generate substantial improvements across all areas, with treatment research holding the most potential.

Other than the aforementioned achievement, progress has also been made in various other fields of study, with neural networks being one of the most researched topics as they provide a lot of flexibility in problem solving, especially in the field of physical sciences [3]. For example, in a study that focused on predicting groundwater potential in arid and semi-arid environments [4], Yunzhi Chen et al. compared hybrid deep learning and machine learning algorithms—boosted tree (BT), artificial neural network (ANN), deep learning neural network (DLNN), deep learning tree (DLT), and deep boosting (DB)—and identified that the deep boosting algorithm could be used in real-life applications to identify possible groundwater spots and thus take the precautionary measures to protect them. Another study performed by Xin Qian et al. [5] presents the advancements made possible by machine learning when predicting thermal transport properties of solids, which, when compared to more traditional methods, provide a cost-effective way of predicting thermal efficiency, even in multi-scale structural designs.

As seen from all the previous advancements, machine learning has started to gain traction in multiple areas, especially in the last 5 years, spanning from big domains like medicine [1,2] and embedded systems [6,7,8,9] to more niche ones like thermal conductivity [5], 3D printing [10], crowd counting [11,12], security [13], and even human augmentation [14]. One outcome that can be extracted from all of these advancements is that AI tasks are becoming more and more complex, thus creating an application from scratch for each new problem requires more time and effort.

Because of this, libraries or frameworks that encompass core functionalities such as layers, initialization strategies, loss functions, optimizers, etc., have become the main way to develop a neural network application. The usage of libraries also removes the need for defining said components, in turn speeding up the process of designing the network structure and shifting the focus to actual layer design. However, in recent years, the number of such libraries and the similarity between some of them have made it increasingly difficult to decide on which one is better suited for a specific task. As such, the study performs an analysis using several criteria, each of them focusing on important aspects that could play a role in choosing one library over another.

*User-friendliness* was chosen with the intent of highlighting how easily could users work with the defined components to achieve specific network structures, a factor which in turn would speed the development even further by making mistakes more visible.

*Available Documentation* is another factor that influences the design phase through the clarity with which it describes the available components. The concise manner in which this information is presented can be a deciding factor for a wide range of users, especially for less experienced ones. Therefore, it was included.

*Ease of integration* is the last factor included in the design process and is focused on how easy it is for a library to be integrated into a system. The intent of adding this indicator in the analysis was to emphasize the faster development and, in turn, the longer refinement phase, which can be achieved with an “easy to integrate” library.

Regarding the training phase, the *Overall Training Time factor* indicates how each library performs on a given task, provided they have the same hardware specifications. The inclusion of this one was necessary as it directly influences how long the training cycle takes to complete, which can make a difference during development.

*Overall Accuracy* is another key indicator that highlights the influence the implemented library has on the accuracy of the results. The reason it was included in the analysis is due to the importance of accuracy in neural network systems, as it decides if the application performs as expected or needs to be redesigned.

The last criterion is *Execution Time During Evaluation*. This factor pertains to the time it takes for the system to produce the result and can heavily influence the viability of the system on given tasks, hence its inclusion was a necessity.

Regarding the chosen libraries to be compared, the bases onto which they were selected are the apparent similarity between them and their popularity among the users. As such, PyTorch and TensorFlow were picked due to their similar approach to designing neural networks and their popularity, being the top two libraries in the field of artificial intelligence.

To emphasize the characteristics of each library, a complex task was created, starting with the work of Zbontar and LeCun [15] in the depth estimation field. Their study focuses on a Siamese Neural Network that, when trained, outputs a depth map extracted from a stereo image pair. In our case, the algorithm starts by generating a set of 9 × 9 image pair patches from 200 stereo images contained in the KITTI Dataset 2015 [16]. Said patches are organized in positive and negative sets which are then normalized by subtracting the mean and dividing by the standard deviation and are used in the network during training in a supervised manner [17]. The architecture of the network is composed of four convolutional layers with a kernel size of 3, 64 output channels, and padding enabled. Each layer is followed by a rectified linear unit (ReLU), the only exception being the last convolutional layer. When an input batch is received, the left and right images are taken through the layers, with their results then being unified using a cosine similarity layer. During training, the library to be used can be chosen dynamically and the network’s parameters are initialized [18] using the Xavier Uniform. The map obtained from the layers is then compared with the ground truth of the set to obtain the loss value of the example. This process is done through a custom hinge loss with an error margin set to 0.2 [15]. The resulting loss is then used in a stochastic gradient descent optimizer with a momentum set to 0.9 and a learning rate of 0.002 for the first 10 epochs and 0.0002 from epoch 11 onwards. The network is trained for a total 14 of epochs, with each epoch processing the whole dataset of around 17 million examples in batches of 128. The accuracy of each epoch is calculated by determining how many positions satisfied the error margin of 0.2.

During evaluation mode, whole images are given to the network and the resulting map is computed for each disparity under consideration, with the actual output then being the positions where the most similar pixels were found for each position. To calculate the error rate, the output was iterated over and each position where the value differed by more than 3 was counted. The result was a percentage between the counted values and the total number of pixels in the image.

### Novelty of the Proposed Method

The comparison between the libraries revealed that with the six criteria (user-friendliness, available documentation, ease of integration, overall training time, overall accuracy, and execution time during evaluation), the differences between multiple libraries can be highlighted and used to decide which library is better suited for the required problem.

Based on all the above, the study extends our previous work [19] and proposes a new methodology of comparing libraries, which can be extended to a larger number of neural network development platforms and be used as a guideline for the users in this domain. Thus, the novelty of the approach is that it offers a guideline based on six criteria (user-friendliness, available documentation, ease of integration, overall training time, overall accuracy, execution time during evaluation), that, in the case where most of the external factors that could influence the comparison are removed, covers not only the aspects related to performance (accuracy and execution times), but also important aspects involved in the development phase of the solution. In the end, this results in more time being allocated to testing the solution and fine tuning, rather than in development.

Moreover, using the previously mentioned guideline, the paper also proposes a methodology for comparing neural network libraries more efficiently, and in return allowing users to choose the library that is best suited to their needs.

Lastly, as a result of applying the guideline and the methodology described above, the study also proves that the choice of library does indeed have an influence, both from a performance perspective as well as a design perspective, and because of this, when developing a solution that utilizes a neural network, the library in which the network will be developed should be a factor worth investing time into.

## 2. Theoretical Context and Study Objectives

A neural network is a “Machine Learning” algorithm that focuses on generating desired outputs by learning certain patterns from the inputs it is given. The general structure of such a network consists of approximations of multiple parameterized functions, with each such approximation being created by taking the inputs through the network and updating the weights of the function described in each layer. The final result is a network capable of reaching the desired outcomes by defining an interconnected structure similar to how real neurons are connected in the brain via synapses [20].

There are multiple ways through which the process of learning can occur, and all of them focus on the way the inputs and outputs interact with the “weights” defined in each function. For example, in the approach of “supervised learning” [17], the network is trained using multiple mappings of inputs to desired outputs. The process involves updating the weights of the network by obtaining an output from the network and comparing it to the desired output, with the difference between the two then being added to the general loss of the system. In doing so, the network learns to extract the features from the input which leads to the desired output. This learning method usually leads to good results, however, it is highly dependent on how varied the training set is, as similar pairs will lead to the network, learning to tune to them specifically, leading to undesired outputs when a different set would be given.

Another example is the “semi-supervised” approach where the same set of input–output pairs is given, along with a larger set of inputs without a mapping (“unlabeled”) [21]. The labeled part is usually created manually from the unlabeled set, and using them in conjunction leads to considerable improvements.

Lastly, there is the “unsupervised” approach [22], where the input set is completely unlabeled and the networks learn to find patterns in it, rather than classify them.

Other than manipulating the input, a network’s behavior is also influenced by the update strategy used on the weights. Without any strategy, the weights would be updated too little, leading to no improvements, or too much, leading to them becoming too big (“exploding gradients”) or too small (“vanishing gradients”). In either case, the resulting input will have no impact on the network, again leading to no improvement. These strategies are known as optimizers, and they control the rate at which the weights are updated to avoid the aforementioned problems [18].

With that in mind, the main objective of the paper is to perform an analysis of important aspects that arise during the development of neural network applications by comparing two of the most popular libraries, those being PyTorch and TensorFlow. The intent is to determine if said aspects can be used to highlight their differences, while also finding if the choice of a library can impact the overall performance of the system either during training or design. Additionally, to ensure that the only influence on the system is the said criteria, a secondary objective is also defined—the removal of all external factors that could influence the comparison.

To achieve the above objectives, the sections that follow present examples of applications that use various libraries to define their neural networks. Next, comparison factors that can be used during evaluation are identified, and then a short description of two of the most popular libraries, along with their strong and weak points that arise during the actual side-by-side comparison, is presented.

## 3. Applications Built Using Neural Network Libraries

Many neural network applications employ the use of libraries to define their models, with the most popular being either TensorFlow or PyTorch. As such, the areas where the two libraries can be utilized are quite vast and diverse. For example, a study regarding the classification of different weapon types [23] defines a new network model created using layers and functions from TensorFlow. The resulting model consists of 25 layers, with the output being a vector and each entry corresponding to a weapon category. Regarding the accuracy of the developed system, using their method, the authors obtained an accuracy of 98.40% which proved the efficacy of the system when compared to older methods such as VGG-16, ResNet-50, and ResNet-101 models. On the same note, another study done by B. Sai et al. [24] trained a CNN to detect threatening objects such as weapons in images by using the Tensor flow Object Detection API. The next study also uses TensorFlow, but this time with the intent of defining a method to assess the quality of given images, without prior references [25]. The created model uses 6 networks in parallel, all created using pre-existing models—VGG16, ResNet50, InceptionV3, InceptionResNetV2, DenseNet201, and NASNet-Mobile—with their outputs being weighted using global average polling layers and used to calculate the final quality score. The obtained results were then compared by the authors with the standalone trained networks, as well as a multitude of other implementations, with the new method offering competitive accuracy. Another application employs the use of TensorFlow to detect surface defects on ceramic tiles [26] by defining a network model composed mainly of four convolutional layers, each followed by a max pooling layer. The output of said structure is a classification of the type Cracked/Not Cracked, indicating if the provided image contains cracked tiles or not, and, with the comparison against other methods, the resulting application provides an accuracy of 99.43%, an improvement of 1.47% compared to the closest second. Similarly, TensorFlow was also used to develop a system capable of identifying patterns in GPR (Ground-Penetrating Radar) images [27]. In the paper, Yuanhong Li et al. modified a YOLO v3 neural network and obtained a system that can offer competitive accuracy, while also being fast enough (~15 fps) to be used in a real-time environment.

Regarding applications that use PyTorch, an example of its usage can be found in the work done by Xabier Cid Vidal et al. [28] that compared the library against other frameworks with the intent of highlighting the importance of deep learning techniques in the field of high-energy physics. To be more precise, the authors performed an analysis of the accuracy of both traditional and deep learning methods to distinguish between signal and background in particle colliders, with their results showing that improvement can be achieved by using neural networks. Another example of usage can be found in the work done by Baohua Yang et al. in [29], where they use PyTorch to develop a neural network that is capable of correctly estimating the nitrogen content in wheat via images. The resulting model mainly consists of 5 convolutional layers and 3 pooling layers situated at the beginning and end, and achieved an accuracy of 97.5% on the calibration set and 86.1% on the validation set, proving that it represents a good option when estimating the level of nitrogen in wheat. On a similar topic, PyTorch was also used in a study that aimed to develop a deep learning algorithm that could detect potato disease based on images [30], the intent being a reduction in the number of chemicals utilized. In it, three network models were tested—GoogleNet, VGGNet, and EfficentNet—on a set of various datasets about the stages of the early blight disease, with EfficentNet being the most suited model for real-world usage.

With all the above, each study provided competitive accuracies compared to methods that are specific to their domains, however, in most cases, only the chosen libraries were used, leaving the possibility of further improvements by implementing said models in a framework that is better suited for the task. Comparisons between frameworks have been made with this goal in mind, one such example being the work of Gurucharan M. K. in TensorFlow vs. PyTorch—Convolutional Neural Networks (CNN) [31]. In it, the author performed a comparison by using the Fashion MNIST dataset and LeNet 5 architecture, in the end, obtaining a leading accuracy of around 2% in favor of TensorFlow. However, most comparisons focus only on certain aspects of the libraries and do not take into account how the said library would integrate into an existing system or how suited it is for a specific task.

## 4. Comparing Neural Network Libraries

To eliminate as many external factors as possible so that there is minimal external influence on the comparison between network libraries, three main prerequisites have been defined. Starting from how the input–output pairs are formatted, the system should ensure that the same dimensions are given to each implementation of the network. The same dataset should also be used on both networks to generate said pairs and to ensure that no advantage can be obtained by using different sets. Regarding the second prerequisite, the implemented networks should have the same architecture, as well as the same optimization strategies, to ensure that the only factor that could influence this step is the internal implementations of the libraries.

Lastly, the developed systems should be evaluated on the same hardware so as to not influence the training times and overall results of the analysis. Achieving these prerequisites will ensure that the only factors which could influence the outcome of the analysis are the ones that are under observation, which are presented below.

A. *User-friendliness*

User-friendliness represents the first aspect to consider when developing a neural network. While not as important to an experienced user, having the library be as user-friendly as possible means a quick learning curve and the possibility to easily define complex code that is readable and understandable. As such, in practice, user-friendliness is gauged by how well users can identify key functions such as loss strategies, optimizers, and network layers without having any prior experience with the library at hand. How much meaningful information eventual errors give also falls under this category, as they can help in determining the cause of the problem more easily and consequently solving the subsequent issue faster.

B. *Available Documentation*

Documentation support is another aspect tied to the “Design Phase” of a neural network and, similar to user-friendliness, it can have a significant impact on development time. This aspect represents the documentation that has been made available online for any user of the library and is the gateway to access all the features that the framework offers, as well as the first place to search when errors occur. With that in mind, a well-documented library will maximize development time by significantly reducing the learning curve and transitioning times, and in turn creating a positive experience for both new and old users.

C. *Ease of Integration*

Another factor important to the “Design Phase” is how well does the library in question integrate with the platform. During development, an easy to integrate library will mean that minimal time is spent setting up the environment, ultimately allocating more time to actual network design. In doing so, using the library will prove to be more efficient in terms of implementation speed, thus reducing implementation costs and effort dramatically.

D. *Overall Training Time*

This aspect refers to how much time is spent during the training of the neural network. Ideally, a library should have minimal impact on training times, therefore allowing for more time to be spent debugging the application in case the results are not ideal. As such, to be able to compare this aspect on multiple libraries, the same network architecture and data flow should be implemented in all instances, and the problem which is being addressed should have a high-enough complexity to ensure that results are comparable. Moreover, data should also be collected from multiple checkpoints, ensuring consistency across them.

E. *Overall Accuracy*

Overall accuracy is the next aspect to be taken under consideration and represents the whole purpose of building a neural network, that being to provide sufficiently accurate results, such that the initial problem is covered for most of the possible inputs. As such, the goal of any neural network library is to provide ways to maximize said accuracy to the best of its abilities. Due to all the above, this category was deemed as one of the most important in the analysis.

F. *Execution Time During Evaluation*

The last factor to be included in the comparison is execution time, and it refers to the time it takes for results to be generated during evaluation. The reason why the addition of this aspect was necessary pertains to its applicability in real production environments, where a difference of a few seconds can be the deciding factor between an accepted solution and a failure.

## 5. PyTorch

PyTorch was launched in 2016 and revolves around the idea of keeping the API simple, allowing it to be quickly modified and kept up to date with the latest trends in the field of AI [32].

It does so by adhering to three main principles, of which the first is the method through which its functions are defined. The framework defines all its components in a pythonic way, the intent being to make it easily usable to users who are already familiar with the Python programming language.

On the same topic of simplicity, the fact that components are defined through interfaces is the second principle of PyTorch. Through this, the complexity of defining and using components is masked by simple initializations and function calls, leading to a clearer identification of core neural networking concepts and therefore an easier learning curve.

The last principle of the framework is that a simple design is better, even when some performance is sacrificed, as a more complex design could lead to the integration being more complex and losing performance in the long run. This approach also has the added advantage of being easier to maintain, resulting in a quicker response to eventual problems that may appear either in the framework or outside.

## 6. TensorFlow

The first version of TensorFlow was released in 2015 and aimed to bring together multiple libraries into a cohesive package that could be used to solve a variety of machine learning problems [33]. This led to a lot of flexibility when designing a network model, as there were libraries such as Contrib and Keras which could be used to define the layers. For an experienced user, the framework offered all the necessary tools, however, the fact that multiple libraries were brought together also meant that some interactions were more complex and harder to understand. An example of such an occurrence was the need to define separate codes to be able to train the network, making mistakes harder to spot and fix. Due to this, the framework was originally seen as not so user-friendly, however with version 2.0, released in 2019, the previous shortcomings were resolved [34].

This new version brought major design changes, starting with the removal of some libraries with the intent of eliminating the confusion created in some parts of the system. To further bring the library more in line with the competitors, the previous two-step process used during training was simplified, with the second step being handled internally, making the process more traceable. Further extending the idea of simplifying interaction, the new version also added an easier instantiation process, based on the pythonic way of user interfaces such as classes to define core components.

Other than fixes to previous problems, TensorFlow 2.0 also added new features such as the TensorFlow Datasets module which provided an easy way to feed data to the network and a new abstraction of the created model during runtime, which meant that a network could be trained on a local GPU or even a multi-GPU environment without any modifications needed.

With all the changes, TensorFlow 2.0 became quite a popular choice among the experienced and inexperienced members of the community, with it offering very good performance while also having an easy-to-use interface, which helped to greatly reduce the learning curve.

## 7. Detailed Design and Implementation

The concepts and requirements presented previously served as the basis for which the actual system is built, with each implemented concept contributing to the accomplishment of said requirements and being represented in the actual algorithm by different components. Furthermore, to eliminate any possible alteration of the results—coming from different implementations of the same solution (one for each library) —the developed test platform presents a single solution with the option of dynamically switching libraries, thus the only components that differ are the ones related to each said library. These shared components, along with the components resulting from the implementation of specific functions, are interwound to form the two main stages of the system (*Training and Evaluation*) and are described in the sections that follow.

### 7.1. Overall Architecture and Control Component

To abide by the various requirements, the system was implemented in such a way that the libraries could be interchanged, allowing it to be run on the same hardware without requiring another project to be created. While this approach makes the project easier to manage, it also adds the complexity of having to manage two network libraries on one project, which means that a central control component is required.

**Figure 1:**
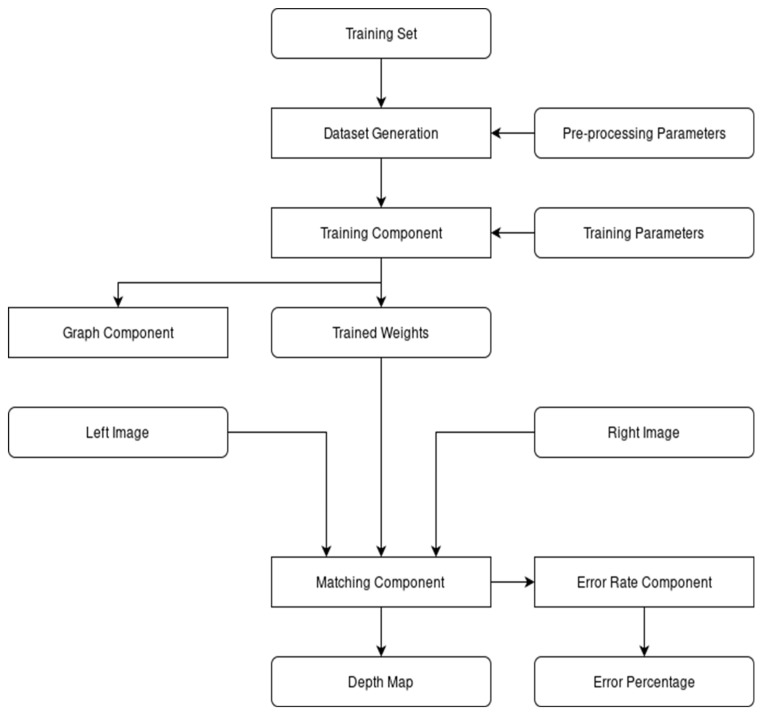
The architecture of the system.

The entire purpose of this central element is to initialize and control the other components of the system via parameters. With it, the user can control what library to use during training or evaluation, which of the two stages the system is in, as well as control very specific learning variables, such as the learning rate or batch size. The following diagram, presented in Figure 1, describes the general architecture of the system.

### 7.2. Dataset Generation

**Figure 2:**
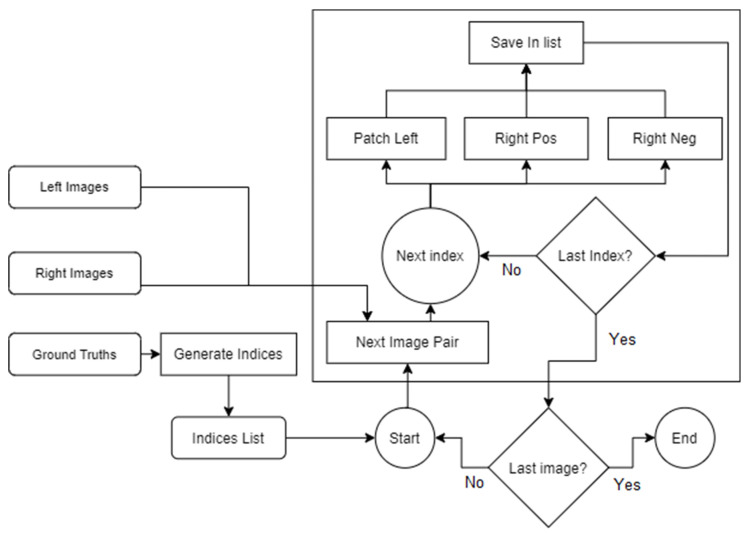
Dataset generation.

Regarding the second requirement, training images are taken from the KITTI 2015 dataset [16], which consists of 200 stereo images along with their corresponding true depth. To not limit the choice to only one training set and to make sure that the system always receives the dataset in a consistent form, the system provides a translation layer that was used to transform the data into smaller, same-sized patches, maintaining accuracy at the cost of performance. Figure 2 describes the final implementation in more detail by presenting the flow of data, as well as illustrating how the various algorithms are interconnected.

The inputs for this stage are represented by the left and right image pairs taken from the KITTI dataset, as well as their corresponding ground truth images. By processing them through the generate set and generate indices components, the array of patch examples is created, with one example containing the left patch, its corresponding patch from the right image (positive right patch), and its negative counterpart that provides an erroneous pairing. Besides the patches, another value is added and represents the corresponding image number from which the patches were taken. It was created as a side-effect from the optimizations decided for calculating the mean and standard deviation and has the role of indicating which precomputed values should be used on the patches.

Implementing those steps also had the effect of multiplying the total number of examples in the input dataset, from the original 200 to approximately 17 million. Doing so allowed for better control over how they are passed to the network, as well as offering more diversity and, consequently, better generalization.

### 7.3. Network Architecture

As previously stated in Section 2, comparing the two libraries first requires the system to provide the same neural network architecture and the same input data format. As such, the defined network is inspired by the work of Zbontar and LeCun [15] in which they described an architecture meant to be used in in-depth estimation applications.

Following the realization of the previous algorithm, its output serves as the training dataset for the neural network component as it provides the input–output pairs necessary in the learning process. This set of examples, however, cannot be trained on at once, as doing so would require a lot of hardware resources and mean that updates would be done only at the end of the dataset, increasing the number of epochs with it. Instead, the process of obtaining a predicted output is split into two main parts, with one of them focusing on organizing the received examples into smaller batches such that they fit the constraints imposed by the computer and increasing the frequency of updates, while the latter focuses on processing said batches through the layers of the network with the intent of obtaining an output and learning from it.

**Figure 3:**
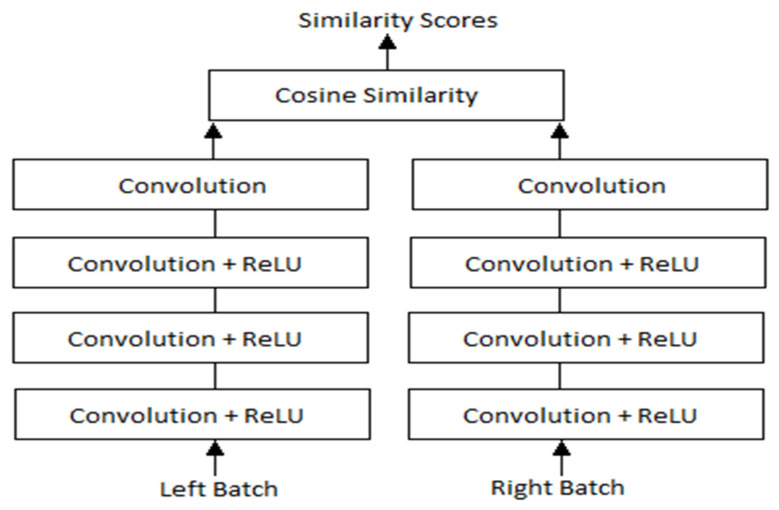
The architecture of the network [19].

Regarding the layers, some modifications were done during implementation to offer a simpler understanding of the network. In [15], Zbontar and LeCun stated that their implementation split the cosine similarity layer into two parts, with a normalization layer being used as the end for the sub-networks, while a Dot Product would serve as the end of the network. They suggested this change as it would offer greater computational speeds, however, for the project at hand it offered marginal advantages, leading to the option of keeping the cosine layer as is to be chosen. By doing so, the overall simplicity of the network would remain intact, while also being less error-prone due to it not being split into multiple parts. Those changes can be seen in Figure 3, which describes the overall network architecture.

As Figure 3 suggests, the left and right batches of examples constitute the inputs for the network, which are propagated through the four convolutional layers and joined at the end by the aforementioned cosine similarity layer.

### 7.4. Loss and Learning

This component is responsible for using the network’s output to compute the loss, with the intent of performing the necessary parameter updates through it. The method through which this is done is by computing the gradient of each parameter and using it to modify its current weight, in turn learning how to better extract features [35]. During implementation, the algorithm described above was split into two main parts, with one handling loss computation, while the other focused on computing the gradients and updating the network through them.

**Figure 4:**
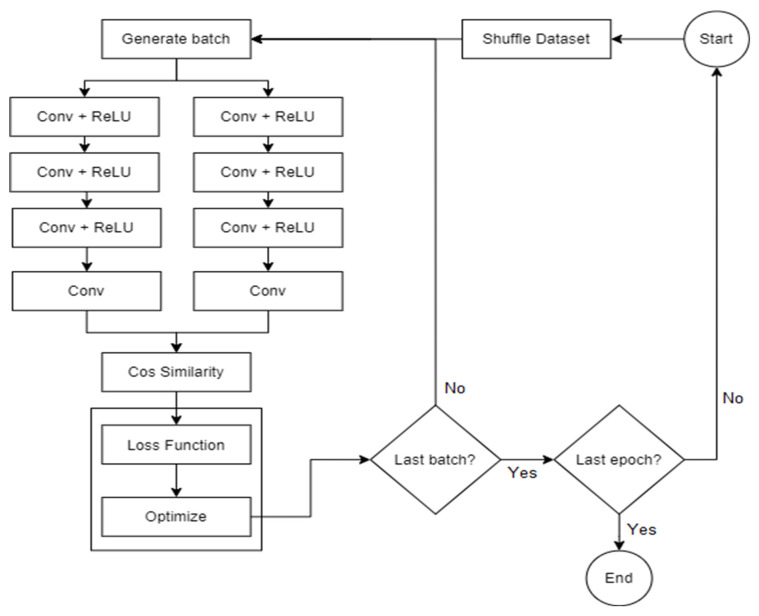
Training flowchart.

The general steps performed by the two algorithms are described in more detail in the following sub-sections, with the first one detailing how the loss is generated and what parameters influence it, while the second consists of the various techniques used during gradient computation and how the parameter updates are performed. The Figure 4 training flowchart also offers a description of said steps in the form of data flow, starting from the moment the batches are created, up to the point where the network’s parameters are updated and have the intent of offering a better understanding of how data are transformed and how each component is involved in the process of obtaining the result, as well as the process of learning.

#### 7.4.1. Loss

The implementation for this method starts in the same function where the batches are forwarded to the network, as its output is needed to compute the loss. When the result is received, the function forwards it to the loss algorithm corresponding to the chosen library, which computes the necessary value by comparing each pair of positive and negative examples, with the cases where the positive is scored higher than the negative by at least 0.2 being classified as valid and receiving 0 loss, with the others being given the loss score based on how big the difference is. In the end, the resulting values are summed up or averaged depending on the user’s desired strategy. The steps required to perform the constraints are described in more detail in Algorithm 1.
**Algorithm 1: Loss Computation**arguments: input_data, saved in memory {margin, reduction} pos = get_pos(input_data)                neg = get_neg(input_data) total_error = max(0, margin + neg – pos) **if** reduction is mean **then**  **return** mean(total_error) **end if** **return** sum(total_error)

The margin, as well as the reduction, are two parameters saved in memory and specified by the user, with the first representing how much higher the positive example should be scored compared to the negative, and the latter determining what strategy to use to obtain the total loss value. Following this, the positive and negative example pairs are extracted from the input, with the error for each pair being computed using the modified hinge loss. In the end, the total loss is obtained using the specified method and is returned as a single value to the calling function.

#### 7.4.2. Backward Propagation

With the previous component’s output received, this method’s implementation starts with backpropagating from the total loss by taking its values and computing the gradients of each parameter by taking into account its corresponding weight. As the name suggests, this process is done in a backward manner, meaning that starting from the loss, gradients are generated from the last layer up to the first, with each intermediate result serving as the basis for computation [36]. Following this, the weights for the parameters are updated and the process repeats itself, starting from computing the batches until the required number of epochs is hit. The following pseudocode details the steps are taken both for obtaining the processed network result and for the algorithm.

As Algorithm 2 shows, the first step in the implementation is to process the batches through the network, the intent of it being to obtain the result that is necessary for computing the loss. With it calculated, the next step is to forward the result to the loss function to generate the total value, necessary for the last step which is calculating the gradients through backpropagation. At the end of the algorithm, the weights are updated through the gradient descent method and represented by the “optimizer.update” step. A check was also done to test the current epoch number, with the intent of modifying the learning rate of the optimizer.
**Algorithm 2: Optimization function**arguments: batch_left, batch_right, epoch result = network_forward(batch_left, batch_right) loss = CustomLoss_forward(result) grads = backward(loss) optimizer.update(grads) **if** epoch == 11 **then**       optimizer.learning_rate(0.0002) **end if**

### 7.5. Feature Extraction and Disparity Generation

**Figure 5:**
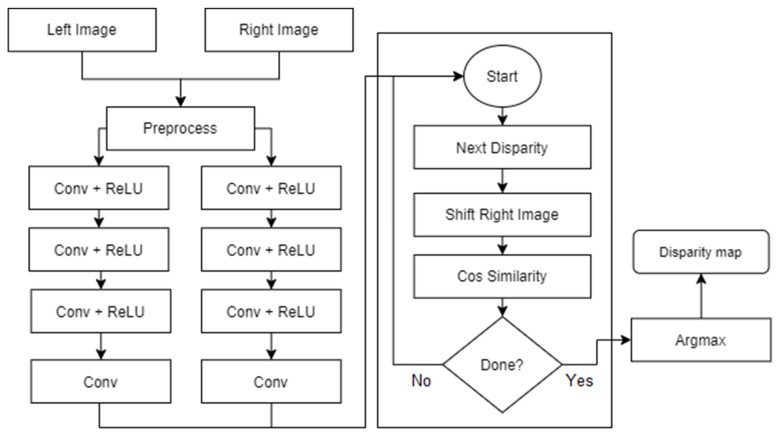
Evaluation flowchart.

This component represents most of the “Evaluation Phase” of the project and is responsible for the extraction of features and computation of the cost matrix, as well as the generation of the depth. The three responsibilities each have their separate function and closely work together to provide the desired results. Figure 5 offers a description from the perspective of dataflow and aims to illustrate the ways through which the various algorithms communicate.

The method starts by receiving a pair of stereo images as input, which are then normalized by subtracting the mean and dividing by their respective standard deviations. The algorithm continues by forwarding the normalized images to the network using the forward functions. They are implemented in the same way as their training counterpart, with the only difference being that the resulting two outputs are not given to the cosine similarity layer, but instead are returned directly. This is then followed by a For loop that cycles between d disparities (specified by the user), with each iteration shifting the right features and the resulting empty spaces being filled with the opposite values taken from the left features. At the end of the loop, the newly formed array, as well as the left features are forwarded to the cosine similarity layer, with the output of it being an array of similarity scores which is saved to the corresponding disparity in the cost matrix.

The resulting matrix contains a vector of size d at each (i, j) position, with each value corresponding to the similarity score obtained from the pair of left–right features. This array serves as input to the searching strategy, which in this case is simply a search for the position of the highest value in the disparity vector on each (i, j) position. The output of it represents the final depth map, with its containing values representing the disparity with the highest similarity between the left–right feature pair.

## 8. Comparing PyTorch and TensorFlow

With the steps described above defined, the system was evaluated on a computer running Windows 10 Pro version 20H2 and used PyCharm Professional Edition with Anaconda plugin, version 2020.1.3 as its IDE.

Regarding hardware specifications, the components used during implementation and testing were the following:

- **CPU**: AMD Ryzen 7 1700X, 3.80 GHz
- **GPU**: ASUS GeForce GTX 1070 Ti A8G, 8 GB GDDR5
- **RAM**: 16 GB 3200 MHz
- **STORAGE**: 500 GB SSD

Lastly, the tests were performed by carefully analyzing each framework, following the guidelines described in Chapter 2.

### 8.1. User-Friendliness in PyTorch vs. TensorFlow

**Figure 6:**
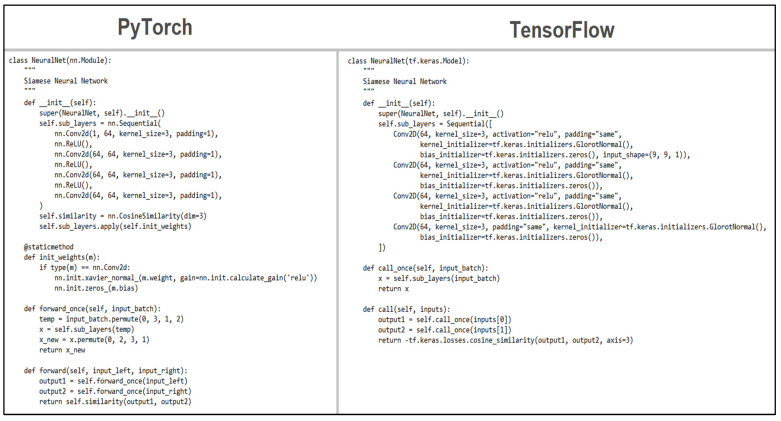
Network architecture comparison.

Following the diagrams for the components defined in Chapter 6, a multitude of functions and classes were created using each library, starting with the loss and the network architecture. For the network, both libraries allowed the creation of layers by extending a default module and using its predefined functions, access, then being done through an interface with two methods—one for data propagation and one for initialization. Figure 6 shows a side-by-side comparison of the written code in both libraries.

**Figure 7:**
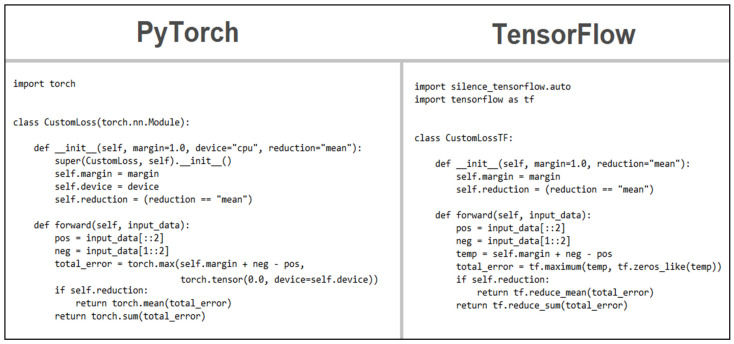
Loss function comparison.

Regarding the loss algorithm, as it was a custom one, none of the libraries offered any closely related function to the required structure and inputs but instead offered ways to define the function in a way similar to how the network was defined. In all instances, the implementation of the algorithm was very similar, with the image that follows, presented in Figure 7, offering a visual comparison.

In the case of the optimizer, the libraries opted for different methods, with PyTorch instantiating the optimizer through a predefined class, while in TensorFlow, even though it also offered an array of default optimizers, none fit the required structure, hence a custom one had to be created by extending a default class.

**Figure 8:**
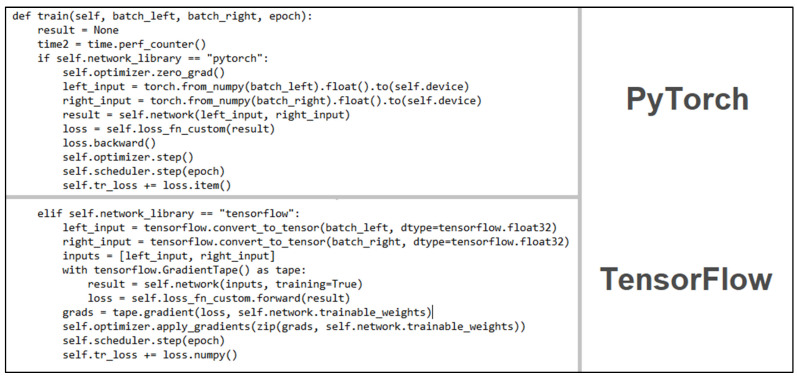
Training flow.

Lastly, bringing together all the aforementioned components to create the training step also presented some notable differences. While each function had a clearly defined role and could be easily traced in both cases, the function calls themselves presented a more complex implementation in the case of TensorFlow, with the gradient generation step being particularly less clear to understand. Figure 8 presents the aforementioned flow in greater detail.

### 8.2. Documentation Support

During development, the documentation of each library was consulted with each function usage and each time errors occurred due to erroneous implementation. For each instance, both libraries offered clear and concise information regarding the usage of modules and eventual solutions to problems. The only corner case where a solution could not be found was during the optimizer implementation in TensorFlow, where no module was suitable for the given structure. This case, however, was dismissed as the documentation provided suitable alternatives, such as extending the default module with a custom implementation.

### 8.3. Integration with the Developed Platform

As the system was created in such a way that the two libraries could be interchanged, their integration needed to be done in such a way that they did not interfere with each other. To accomplish this, the two implementations were separated into packages, and control variables were used to determine what library to use. As both PyTorch and TensorFlow offered easy-to-use interfaces, the integration step was completed with relatively low errors, with the only notable occurrences being during the installation of both libraries, where there were some incompatibilities between them. These incompatibilities were generated due to the external requirements presented by TensorFlow, mainly the presence of a local CUDA installation, which interfered with PyTorch’s included installation via pip. Attempts were made to install a local version; however, the available windows version was lower than what TensorFlow required, leading to the inability to use it. In the end, the solution was to point to PyTorch’s CUDA installation, however, it took a lot of trial and error to reach it as neither documentation had any information regarding this issue.

### 8.4. Training & Execution Times

Following the general requirements described in Chapter 3, timestamps need to be collected at critical points in the system to determine if the two implementations had any effect on the overall performance. As such a suitable place for their placement needed to be found to monitor the changes as closely as possible, the decision was made to place them at the points where the dataflows for each library merged, as they were the closest points that could offer comparable data.

With all the above, Table 1 offers a visualization of the collected data, with *Total training time* representing the total time elapsed for the network to be fully trained on the given dataset, the *Average epoch time* being the time it takes for the network to process the training set once, *Network and Optimization time* describing the time it takes to finish the optimization stage starting from the initial input, and *Execution time*, which is the actual time it takes for a result to be reached during evaluation.

**Figure 9:**
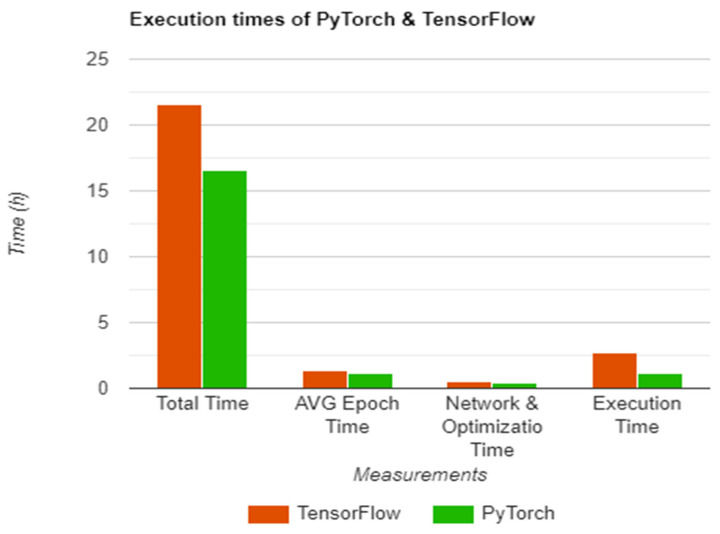
Execution times of PyTorch and TensorFlow chart.

As seen in Table 1 and Figure 9, there are a few differences between the registered performance of each library, with an average difference of 25.5% in training mode, which can be further broken down into 25.5% for total training time, 26.1% in average network time, and 25.1% during the network training and optimization step. Similar results were obtained during the evaluation step, however, on a larger scale, with PyTorch being faster by 77.7%.

These results show that, at least for the training step, the difference is consistent with each checkpoint, leading to the conclusion that the internal dataflow of each library is determining this behavior, with how the data are handled inside the network layers being a deciding factor. Considering that the above table presents a series of access times taken throughout the implemented system, a comparison was also performed against the work of Jure Zbontar et al. in [15], where they also presented a series of accessing times related to their implementation. This analysis was mainly performed to ensure that the developed network was behaving within the margin of error, thus ensuring its correctness when compared to the original implementation from [15]. However, before being able to compare the results we first had to solve the hardware differences—Nvidia Titan X vs. our Nvidia GTX 1070 Ti. While the actual hardware could not be sourced, an approximation based on the difference in performance was possible. Seeing as the GTX 1070Ti contains the same number of CUDA cores and memory as the GTX 1070, a direct correlation could be found by following the comparison found in [37], which shows a clear performance advantage of approximately 84%. With that in mind, the times presented by the authors in [15] were at around 0.34 s for no stereo method, which, translated to our case, would be at around 0.62 s. Those times were performed on a video card with more memory capacity, which meant that no data separations had to be made. In our case, however, we could not perform the cosine similarity operation normally as there was not enough memory to hold the images, and thus we had to optimize our memory utilization method by shifting the right image by a d amount for each disparity d under consideration. This meant that the network could even be run on a video card with 4 GB of RAM, at the cost of time, as the shift operation took more processing resources. With all the above, we measured the time it takes to perform said shift operation—0.002 s for each disparity. In testing we used a disparity of 228, which equates to 0.47 s being lost. Adding those to the 0.62 s that were previously obtained, results in 1.09 s to obtain a depth map, or 7% better than what was obtained in PyTorch. This shows that while in terms of execution times, the framework utilized by Zbontar et al. [15] performs better, the difference is small enough to validate the PyTorch implementation.

The same conclusions can be drawn for TensorFlow, with the addition that some incompatibilities in the implementation, which pertained to the method used in transferring the network results to their respective matrix slices, also added to the difference.

### 8.5. Accuracy during Training & Execution

Besides the checkpoints used to determine the overall performance impact in the training and execution stages, another set of measurements was performed on the system, this time focusing on the overall accuracy of each stage.

Starting with the training step, a chart was used to monitor the evolution of each epoch, with the obtained accuracy representing how well the system was minimizing the loss through the cycles. Attempts were made to generate actual outputs and calculate their accuracy on each epoch, however, doing so meant that a considerable amount of time would be spent on the training phase, as it required the system to switch to evaluation mode each time a cycle was completed. As such, the actual outputs were generated and tested only during the evaluation step by loading network states corresponding to each epoch.

**Figure 10:**
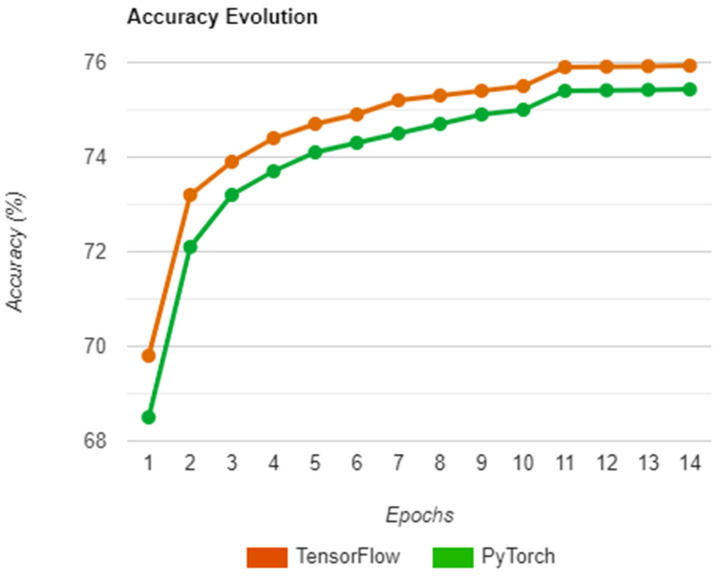
Accuracy evolution over the epochs.

With that in mind, the outcome of a run on both libraries during training is presented in Figure 10 and reveals a similar progress path on each implementation, with both having an initial boost in accuracy and slowly increasing afterward. The reason the accuracy jumps at epoch 11 is due to the training method used, which required a learning rate change from 0.002 to 0.0002 at that specific epoch to ensure that the system does not overfit in the remaining cycles. Regarding the results obtained by Zbontar et al. in [15], we tried to perform a similar comparison to the one in the previous section, however, there were no data that we could corelate with, as the focus of the research paper was on the actual results of the evaluation and not on the training.

**Figure 11:**
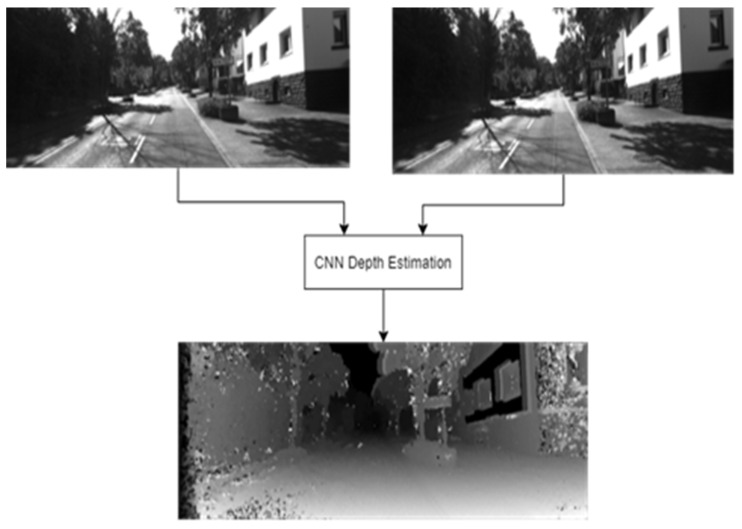
Visual depth map obtained from the network [19].

For the evaluation step, an actual depth map of a pair of examples was used to calculate an error rate value following the guidelines of Zbontar et al. [15]. Figure 11 offers an example of the system’s inputs and output, with the inputs being a pair of stereo images taken from the KITTI dataset [16].

The error rate is an inverse of accuracy, representing the percentage of erroneous pixels in the depth map, and the reason it was chosen over accuracy pertained to the fact that it better represented the output of the system, and it provided an easier method to be calculated:

(1) $$E\left(i,j\right)=\{\begin{array}{cc} 1, & \mathrm{if}\;\left|Map\left(i,j\right)-Ground\left(i,j\right)\right|>=3\\ 0, & \mathrm{otherwise} \end{array}$$

(2) $$error\_rate=\frac{\sum_{i=0}^{h-1}\sum_{j=0}^{w-1}E\left(i,j\right)}{total\_count}\times 100$$

(3) $$average\_error\_rate=\frac{\sum_{1}^{n}error\_rate}{count\_error\_rate}$$

As seen in these three Equations (1)–(3), with the first equation, a matrix of 0 and 1 s is created, with the positions where the pixel value difference between the true depth and the net result is more than 3 being seen as erroneous and therefore being represented by a 1, while the rest is represented by a 0. The erroneous positions are then counted, and a percentage is drawn by comparing the resulting value with the total number of pixels in the result.

With all the above, the measurement was used in two separate tests which aimed to describe the accuracy obtained on a single image pair and a whole dataset. Table 2 shows the results of each test, both being run on the two libraries and having a separate column for each result.

**Figure 12:**
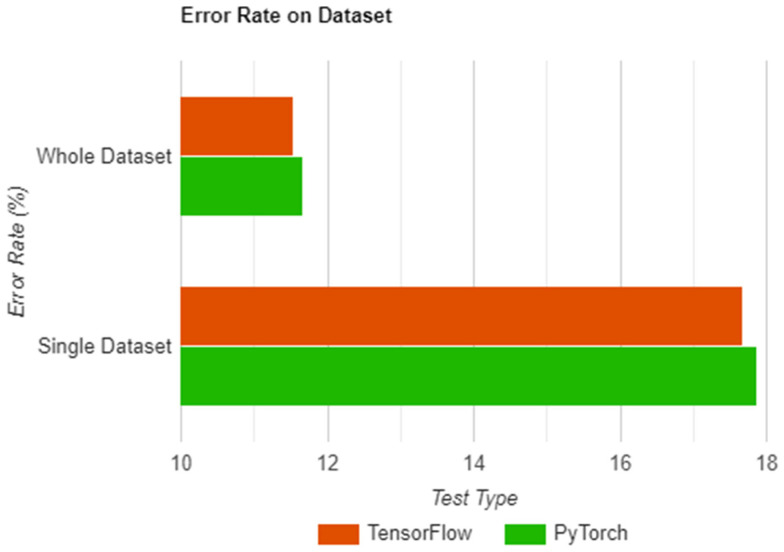
The error rate for PyTorch and TensorFlow chart.

As seen in Table 2 and Figure 12, the results are in reverse of what was obtained in Section 8.4, with TensorFlow having better accuracy both in the single and overall tests. As was the case with the previous two sections, we correlated the obtained results with the results presented by Zbontar et al. [15] in their research paper. This was also one of the reasons why we opted for the use of the “error rate” measurement as opposed to accuracy. As with the previous cases, the results presented in the paper were categorized by the full stereo method (includes post-processing steps) and the non-stereo method which simply outputted the result of their network. As we did not include any post-processing steps in our implementation—our focus was on the network itself and not on the processing steps—we took the result described in the non-stereo method category, which in our case was 15.70% error rate, as we also tested on the KITTI 2012 dataset [16]. With that in mind, the author’s results offer a percentage difference of 12% when compared to our average in PyTorch (17.87%), which, while not disproving our solution, was significant enough to warrant a more in-depth analysis on our implementation. What we found in the end was that Zbontar et al. [15] used dataset augmentation techniques, which were missing from our implementation. This meant our solution was not generalizing as well as it should, as even with the shuffling of the dataset, the same image patches were fed into the network numerous times, resulting in a small loss of accuracy. We then tried to approximate an error rate value stating from the results obtained by Zbontar et al. and by considering no augmentation and no stereo, however, there were too many variables, and a conclusion could not be reached.

**Figure 13:**
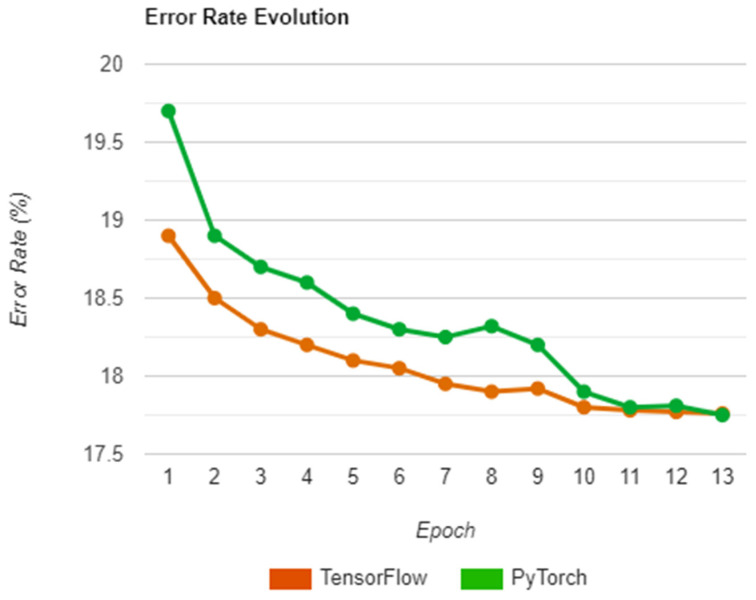
Error rate evolution over the epochs.

Regarding the before-mentioned accuracy progression, a set of tests were also performed on all the saved epochs by loading the network state of each run. These states were then used to compute the mean error rate on the whole dataset and the results were added to a chart, with the intent being to see how each library progressed with generating the depth maps in each iteration. As such, the charts depicted in Figure 13 offer a visual representation of the whole process.

As seen above, the two libraries follow a similar path in terms of error rate, with TensorFlow having better initial accuracy and maintaining the lead until the end with a difference of 1.16%. Moreover, PyTorch also presents a less stable evolution, particularly in epoch 8 where the error rate jumps upwards by a significant amount. This could be due to the random nature of how the input set is shuffled, possibly leading to worse generalization due to similarities between inputs, which could also explain the sudden increase in accuracy that happens afterward as a consequence of better spread.

## 9. Conclusions

With the number of available neural network libraries coupled with the similarities of their offerings, deciding which one is more suited for specific tasks has become increasingly difficult. Because of this, we presented a series of key factors to be used in highlighting the advantages and disadvantages of each library and performed a comparison on two of the most popular neural network frameworks, PyTorch and TensorFlow, with the intent of determining if said factors can be used to differentiate between the two very similar libraries.

The results presented in Section 8 show that a distinction can be made. PyTorch is shown to be focused on the “Design Phase”, with a more user-friendly interface due to it having more functions defined by default—cosine similarity—all while also being easier to integrate in solutions, with a single command to install, no external dependencies, and being slightly below what TensorFlow offers in terms of accuracy during training, with 1.16% difference between the two libraries. Therefore, PyTorch is better suited for learning or tasks where speed is prioritized as it offers a more beginner-friendly experience due to a simpler installation process, easy-to-follow dataflow, and overall easier integration, with higher training speeds 25.5% faster during training and 77.7% faster during execution. TensorFlow, in contrast, is better used by experienced users and in tasks where accuracy takes priority, as it offers more flexibility in both the integration process—any CUDA version compared to PyTorch’s fixed version—as well as in how a network can be defined and trained, with more control over the training flow due to complexity, resulting in a higher overall accuracy.

In conclusion, the choice of the library does influence both design and system performance, and the previously defined criteria of each phase can be used to highlight the different aspects of each library. As such, the provided criteria can thus help users better achieve the requirements of their solutions by highlighting not only the accuracy and computational speed of each library, but also their overall usability.

With all that in mind, the study focused only on two of the most popular libraries and only on one example architecture. Therefore, this research does not represent the majority of use-cases and will extend by focusing on applying the defined criteria on multiple other libraries, spanning over all usage and age spectrums, as well as on a multitude of other network architectures. At the moment of writing this, two more libraries have been identified as potential candidates for testing, being MXNet and Caffe2. They were both launched in 2017 and are similar in how they are used for TensorFlow and PyTorch. Along with the new libraries, a series of other architectures—LSTM (Long/Short-Term Memory), recurrent, and even feed-forward networks—have already been identified as important to research and will be used to further validate the criteria, as well as test for other possible aspects that could arise during the development of neural network applications, such as stability.

## Figures and Tables

**Table 1:** Execution times of PyTorch and TensorFlow [19].

Measurement	PyTorch Time	TensorFlow Time
Total training time	16 h 58 m 51.032 s	21 h 56 m 53.515 s
Average epoch time	1 h 10 m 48.891 s	1 h 32 m 6.028 s
Network & Optimization time	0.0008693 s	0.0011189 s
Execution time	1.1743711 s	2.6666322 s

**Table 2:** Error rate for PyTorch and TensorFlow [19].

Test Type	PyTorch Error Rate	TensorFlow Error Rate
Single image	11.6752832%	11.5439943%
Whole dataset	17.8751599%	17.6685158%

## Data Availability

Not applicable.
